# Nanoscale Investigation of DNA Demethylation in Leukemia Cells by Means of Ultrasensitive Vibrational Spectroscopy

**DOI:** 10.3390/s23010346

**Published:** 2022-12-29

**Authors:** Anca Onaciu, Valentin Toma, Cristian Moldovan, Adrian Bogdan Țigu, Diana Cenariu, Carina Culic, Rareș Mario Borșa, Luca David, Gabriela Fabiola Știufiuc, Romulus Tetean, Ciprian Tomuleasa, Rareș Ionuț Știufiuc

**Affiliations:** 1 MedFuture—Research Center for Advanced Medicine, “Iuliu Hatieganu” University of Medicine and Pharmacy, 400349 Cluj-Napoca, Romania; 2 Department of Pharmaceutical Physics & Biophysics, “Iuliu Hatieganu” University of Medicine and Pharmacy, 400349 Cluj-Napoca, Romania; 3 Department of Odontology, Endodontics, Oral Pathology, Faculty of Dental Medicine, “Iuliu Hațieganu” University of Medicine and Pharmacy, 400001 Cluj-Napoca, Romania; 4 Faculty of Dental Medicine, “Iuliu Hatieganu” University of Medicine and Pharmacy, 400349 Cluj-Napoca, Romania; 5 Faculty of Medicine, “Iuliu Hatieganu” University of Medicine and Pharmacy, 400337 Cluj-Napoca, Romania; 6 Faculty of Physics, “Babes-Bolyai” University, 400084 Cluj-Napoca, Romania; 7 Department of Hematology, “Iuliu Hatieganu” University of Medicine and Pharmacy, 400015 Cluj-Napoca, Romania; 8 Department of Hematology, “Ion Chiricuta” Clinical Cancer Center, 400015 Cluj-Napoca, Romania

**Keywords:** leukemia, 5-azacytidine, Raman spectroscopy, SERS, DNA demethylation

## Abstract

DNA methylation is a crucial epigenetic hallmark of cancer development but the experimental methods able to prove nanoscale modifications are very scarce. Over time, Raman and its counterpart, surface-enhanced Raman scattering (SERS), became one of the most promising techniques capable to investigate nanoscale modifications of DNA bases. In our study, we employed Raman/SERS to highlight the differences between normal and leukemia DNA samples and to evaluate the effects of a 5-azacytidine treatment on leukemia cells. To obtain spectral information related to DNA base modifications, a DNA incubation step of 4 min at 94 °C, similar to the one performed in the case of RT-PCR experiments, was conducted prior to any measurements. In this way, reproducible Raman/SERS spectra were collected for all genomic DNA samples. Our Raman results allowed discrimination between normal and cancer DNAs based on their different aggregation behavior induced by the distinct methylation landscape present in the DNA samples. On the other hand, the SERS spectra collected on the same DNA samples show a very intense vibrational band located at 1008 cm^−1^ assigned to a rocking vibration of 5-methyl-cytosine. The intensity of this band strongly decreases in cancer DNA due to the modification of the methylation landscape occurring in cancers. We believe that under controlled experimental conditions, this vibrational band could be used as a powerful marker for demonstrating epigenetic reprogramming in cancer by means of SERS.

## 1. Introduction

Hematological malignancies such as chronic and acute lymphocytic leukemia, chronic and acute myeloid leukemia, myeloma, and Hodgkin and Non-Hodgkin lymphoma have been reported from early stages of life. Leukemia presents an incidence of almost 500,000 new cases and over 300,000 deaths according to Globocan 2020 statistics [1,2]. 

Genetics play an important role in cancer development, and in the last decades was associated with environmental factors as another hallmark implied in the regulation of gene expression [3]. Epigenetic variations include paramutation, gene silencing, imprinting, X chromosome inactivation, reprogramming, teratogenesis, carcinogenesis, and chromatin modifications [4]. Carcinogenesis involves the inhibition of tumor suppressor genes or the activation of oncogenes through different molecular mechanisms: DNA methylation, histone modifications, nucleosome positioning, and non-coding RNA expression [3,5]. Many of these processes seem to be involved in cellular homeostasis and normal cell development [6]. In humans, the DNA methylation process usually takes place at the cytosine site that precedes a guanine (CpG sites) with the exception of CpG islands in somatic cells [7]. DNA methyltransferases (DNMTs) transport a methyl group from S-adenyl-methionine to the fifth carbon of a cytosine residue and forms 5-methylcytosine (5mC). 5mC cytosine presents a mutagenic potential since it can be deaminated and converted to thymine [8]. The methylated sites, especially the promotor regions, influence the protein-DNA interactions leading to chromatin remodeling and affecting the expression of specific genes [6,9].

Many chemical agents (5-azacytidine, 6-thioguanine, 5-(β-d-Ribofuranosyl) isocytosine, and dexamethasone cytarabine carboplatin) have proved their therapeutic efficiency in hematological-associated cancers by a mechanism involving their interaction with DNA and/or the enzymes involved in the methylation process [10,11].

5-azacytidine (5-azaC) is well known due to its capacity to demethylate DNA by inhibiting the DNMTs activity [11,12]. This compound was firstly developed by Piskala and Sorm [13] and its therapeutic role was studied in acute myelogenous leukemia [14,15]. It was revealed that this analogue of cytosine action implies chromosome breakage [16,17], being considered a mutagen agent [18,19]. 5-azaC is a prodrug and its activation occurs through several phosphorylation steps [20,21,22]. Firstly, 5-azaC is phosphorylated by uridine/cytidine kinase and incorporated into RNA. Secondly, this triphosphate molecule is converted into deoxyribose by ribonucleotide reductase. At this step, 5-azaC can be incorporated into DNA strands during replication and DNMTs covalently bound to DNA are cleaved leading to demethylation and gene reactivation [23,24]. It is important to mention that only 10–20% of 5-azaC deoxyribose is incorporated into DNA while 80–90% remains in its triphosphate configuration incorporated into RNA [20].

Among different experimental techniques that have the capacity to prove nanoscale modification of DNA structure, Raman and its counterpart, surface-enhanced Raman scattering (SERS), has many advantages [25,26,27]. This method is non-invasive, label free, cost effective, and time efficient in generating experimental data that can be further used for diagnosis results [28,29,30]. The scientific literature is abundant in studies describing the use of SERS for the analysis of biomolecules [31,32]. Basically, it relies on the inelastic scattering of monochromatic light by the vibrating atoms within the sample [33]. The major inconvenience that must be overcome for a proper assessment of the experimental data is the improvement of spectral reproducibility [34,35]. In order to avoid these drawbacks, plasmonic nanoparticles were engaged to develop different plasmonic substrates offering stability and reproducibility on microscopic surfaces [29,35,36]. In the case of DNA samples, data assessment is even more complicated since the detection of unamplified genomic DNA presents further challenges such as: low target concentration, very long genetic sequences (thousands of bases in length), and a negatively charged double stranded structure (dsDNA) preventing their direct interaction with the plasmonic nanoparticles, which in most cases have a negative surface charge [37].

In this study, we propose the use of Raman/SERS for nanoscale investigation of DNA samples collected from normal and cancer (leukemia) cell lines. The effect of 5-azaC treatment on the DNA samples collected from leukemia cells has also been experimentally evaluated by means of ultrasensitive vibrational spectroscopy. We used two types of cell lines: a normal one and two leukemia cell lines. The two malignant cell lines were treated with 5-azaC. All three cell lines were processed for DNA extraction followed by Raman/SERS analysis. The SERS experiments were performed by incubating the DNA sample with in-house-synthesized silver nanoparticles, concentrated, and purified by means of the tangential flow filtration (TFF) method, followed by a heat treatment at 94 °C to facilitate the interaction of DNA bases with the plasmonic nanoparticles. The sample preparation protocol for Raman experiments implied the same conditions (DNA incubation step of 4 min at 94 °C). The heating procedure allows a rapid water evaporation and leads to the formation of single stranded DNA (ssDNA) without any structural denaturation with respect to the initial sample. Moreover, the genomic DNA that has both strands separated can bind properly to the plasmonic substrate. By adding this step, the analysis of the modifications occurring at nucleotide level became possible. We also investigated other methods for sample preparation (i.e., mixing DNA samples with silver colloid without heating and drying), but the results were irrelevant (data not shown). 

Our study’s aim is to take advantage of the capacity of Raman/SERS performed on nucleic acid samples to highlight nanoscale modifications in their structure as a result of epigenetic changes and of their interaction with a clinically approved chemotherapeutic agent. Consequently, a better understanding of the cellular mechanism involved in cancer development could be provided.

## 2. Materials and Methods

### 2.1. Colloidal Silver Nanoparticles Preparation

Colloidal silver nanoparticles were synthesized based on a method developed by [38] by reduction of silver nitrate with hydroxylamine. Briefly, 17.2 mg of AgNO_3_ (Sigma Aldrich, Darmstadt, Germany) were dissolved in 10 mL ultrapure water (18.2 MΩ·cm, ELGA Labwater from PURELAB Chorus, Buckinghamshire, UK) and this solution was added carefully by vigorous stirring conditions over 90 mL solution containing 12.7 mg NaOH (Sigma Aldrich, Darmstadt, Germany) and 10.5 mg NH_2_OH·HCl (Sigma Aldrich, Darmstadt, Germany). In order to remove the chemical byproducts and to improve their plasmonic properties, the nanoparticles were concentrated 10× and purified by means of tangential flow filtration (TFF, Pall Corporation, New York, NY, USA) according to a procedure developed in our lab [29], 3 days after the synthesis completed.

### 2.2. Cell Culturing

DAMI Luc2 acute megakaryoblast leukemia cells (CRL-9792) and MEG-1 chronic myelogenous leukemia cells (CML) (CRL-2021) were maintained in RPMI cell culture medium supplemented with 10% fetal bovine serum (FBS) and 1% L-glutamine. LX2 normal hepatic stellate cells were maintained in DMEM 4.5 g/L glucose supplemented with 10% FBS, 1% glutamine, and 1% penicillin/streptomycin. Cells were maintained at 37 °C in a humidified chamber with 5% CO_2_. All reagents used for cell growth were purchased from Gibco (Grand Island, NY, USA). All cells were purchased from ATCC.

### 2.3. 5-Azacytidine Treatments

5-Azacytidine powder (CAS-320-67-2, with 98% purity) was purchased from Tocris (Tocris, Abingdon, UK). A stock solution was prepared in DMSO (Sigma-Aldrich, St. Louis, MS, USA) and stored at −20 °C. Then, fresh working solutions were prepared in a certain culture medium. 

DAMI Luc2 and MEG-1 cells were treated for 72 h with 5-Azacytdine in 96-well plates (1 × 10^4^ cells/well). The cells’ viability rates were analyzed using MTT assay (Boster, Pleasanton, CA, USA). An amount of 10 μL of MTT Reagent was added over 200 μL that contained the cells. The plates were incubated for 4 h at 37 °C in a humidified chamber with 5% CO_2_. The plates were washed three times with PBS 1X and 100 μL of Formazan solubilizing solution was added to each well followed by a 4 h incubation step at 37 °C in a humidified chamber with 5% CO_2_. The plates were analyzed using TECAN SPARK 10M spectrophotometer (Spark^®^, Einhausen, Germany) operating at 570 nm. Statistical analysis was performed using OriginPro^®^2019 version 9.6.0.172 (Academic) software. The as-obtained IC_50_ values were further used as working concentrations, depending on each cell line. The treatment was added fresh, once every 24 h, for three days. These experiments were conducted in duplicate.

### 2.4. DNA Extraction

5 × 10^6^ DAMI Luc2, MEG-01, and LX2 cells per each sample were washed three times with PBS 1X at 161× *g* for 5 min at room temperature. The cell pellet was further resuspended in 200 μL of PBS 1X and stored at −80 °C for at least 24 h. The DNA extraction and purification were performed according to the working protocol provided by PureLink Genomic DNA mini-Kit (Invitrogen, Carlsbad, CA, USA). The 200 μL sample containing cells was thawed at room temperature and mixed with 20 μL RNAse solution and 20 μL of Proteinase K solution, followed by 2 min of an incubation step at room temperature. An amount of 200 μL of lysis buffer solution was added and mixed well by vortexing. The mixture was incubated for 10 min at 55 °C. After the incubation step, 200 μL of 99% ethanol (VWR, Radnor, PA, USA) and 640 μL of the mixture were vortexed and transferred in a purification column provided within the kit, followed by a centrifugation step at 10,639× *g* at room temperature for 2 min. The collection tube was replaced with a new one and 500 μL of washing buffer 1 was added to the purification column and centrifuged at 10639×g at room temperature for 2 min. The collection tube was replaced and 500 μL of washing buffer 2 was added to the purification column, incubated for 2 min at room temperature, and then centrifuged at 17,980× *g* at room temperature for 2 min. Further, the upper part of the purification column, containing the filter, was transferred to a sterile 1.5 mL Eppendorf tube for DNA elution. The elution step was performed by adding 30 μL of DNA/RNA free Ultrapure water (Invitrogen, Carlsbad, CA, USA). The column was incubated for 2 min with the ultrapure water followed by a 3-min centrifugation step at room temperature and 17,980× *g*. The DNA concentration was measured using the Nanodrop 2000c system (Thermo Fisher Scientific, Waltham, MS, USA).

### 2.5. Raman Spectroscopy Sample Preparation and Measurements

For Raman measurements, CaF_2_ glass was used (20 mm diameter and 1 mm thickness, purchased from Crystran Limited, Poole, UK) as the port probe. Firstly, the port probe was cleaned using various solvents (e.g., acetone, ethanol) and ultrapure water and thereafter dried at room temperature for 15 min. Then, the port probe was mounted on a microscope slide and a 2 µL DNA sample (100 ± 5 ng/µL concentration) was poured on top of it. Immediately, the sample was introduced into a pre-heated thermoblock (94 °C) (Eppendorf ThermoMixer C) for 4 min for dehydration and DNA denaturation into ssDNA. After 15 min at room temperature, the samples were ready for measurements. The measurements were performed using a Renishaw™ inVia Reflex Raman confocal multilaser spectrometer (Renishaw plc, Gloucestershire, UK) at a resolution of ~1 cm^−1^. An internal silicon reference was used for calibration. The spectra were recorded using the 50× (N.A. = 0.75) objective. For all the Raman measurements, a 532 nm diode laser was used. The laser power measured relative to the sample surface was ~11 mW, while the integration time was set to 40 s. For each sample, 5 maps with 20 acquisition points for each map were recorded. The spectra were collected at a maximum 50 µm distance from the edge. The spectrograph was equipped with 1800 lines/mm grating and a charge-coupled device camera (CCD). Each final spectrum represents the average of 100 spectral acquisitions. A baseline correction was applied to remove the fluorescence background using the Wire 4.2 software provided by Renishaw (Gloucestershire, UK).

### 2.6. SERS Sample Preparation and Measurements

For SERS measurements, a clean CaF_2_ glass mounted on a microscope slide was used as a port probe. An amount of 1.5 µL of concentrated silver nanoparticles was mixed with 1.5 µL of DNA solution and incubated for 1 h at room temperature. Then, 1 µL of this mixture was put on the CaF_2_ glass and then rapidly placed in a pre-heated thermoblock (94 °C) (Eppendorf ThermoMixer C) for 4 min for dehydration and DNA denaturation into ssDNA. By the end of this process, the plasmonic nanoparticles surrounded by ssDNA strands self-assembled on the CaF_2_ glass and were ready to use for SERS measurements. The substrate was cooled down at room temperature for 15 min. The spectra were analyzed using Renishaw™ inVia Reflex Raman confocal multilaser spectrometer (Renishaw plc, Gloucestershire, UK) at a resolution of ~2 cm^−1^. For spectra recording, the 50× (N.A. = 0.75) objective and a 785 nm excitation wavelength were used. The laser power, measured at the sample surface, was less than 2 mW. The acquisition time was set to 10 s (10 s integration time and 1 accumulation). For each sample, 5 maps with 50 acquisition points/map, acquired at a maximum of 50 µm distance from the edge, were recorded. A 600 lines/mm grating and a CCD were used. Each final spectrum represents the mean of 250 spectral acquisitions. A baseline correction was applied in order to remove the fluorescence background, using the same Wire 4.2 software.

## 3. Results

### 3.1. 5-Azacytidine Treatment Effects

**Figure 1:**
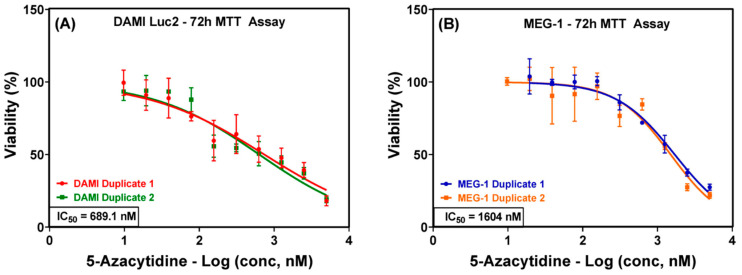
Viability rate and average IC_50_ of DAMI Luc2 cells (**A**) and MEG-1 cells (**B**) treated with 5-azaC for 72 h.

DAMI Luc2 and MEG-1 cells were incubated with different concentrations of 5-azaC (between 9 nM and 5 µM—serial dilutions) for 72 h. The viability rate was determined and a two-way ANOVA test showed the statistical significance between the viability of the treated cells and the control group (Figure 1). Based on the viability rate, IC_50_ values of 689.1 nM for DAMI Luc2 cells and 1604 nM for MEG-1 cells were obtained. Thereafter, the upcoming assays were conducted using the two above-mentioned IC_50_ doses.

### 3.2. Raman Spectroscopy Measurements

**Figure 2:**
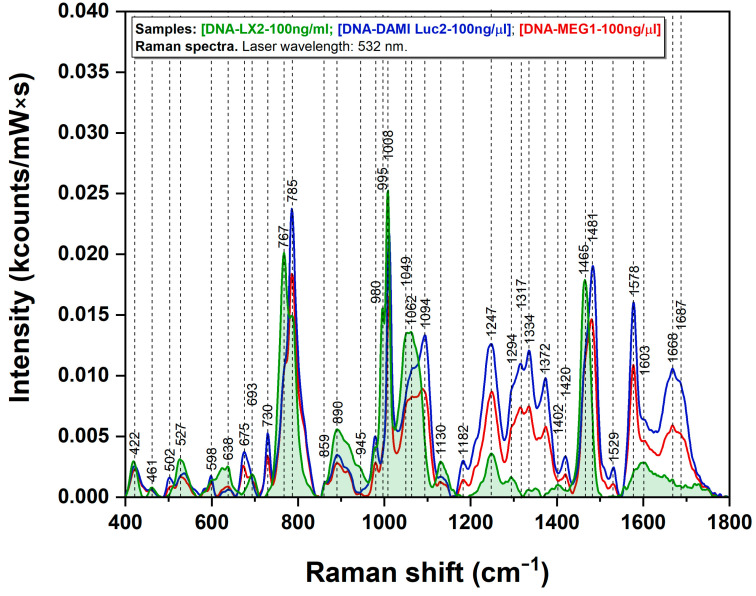
Raman spectra of DNA samples from leukemia cell lines DAMI Luc2 (blue), MEG-1 (red), and normal cell line LX2 (green); recorded using a 532 nm excitation wavelength.

DNA extraction was performed for all cell lines in order to evaluate, by means of ultrasensitive vibrational spectroscopy, the nanoscale structural differences between normal and leukemia cells on one hand, and between leukemia cells before and after treatment with 5-azaC. The DNA sample was poured onto the CaF_2_ glass and prepared for Raman analysis. Figure 2 shows the Raman spectra of DNA extracted from the three cell lines: DAMI Luc2, MEG-1, and LX2. Various major differences can be observed between DNA samples, particularly at 767 cm^−1^, 980 cm^−1^, 1049–1094 cm^−1^, and 1247–1687 cm^−1^. Certain vibrational bands are very intense in the DNA samples extracted from leukemia cells (675 cm^−1^, 730 cm^−1^, 785 cm^−1^, 1247 cm^−1^, 1481 cm^−1^, and 1578 cm^−1^) but are very weak or do not appear at all in the case of normal cells. Some other peaks are more evident in the case of normal cells (638 cm^−1^, 767 cm^−1^, 980 cm^−1^, 1062 cm^−1^, and 1465 cm^−1^). One has to mention that the Raman spectra of the DNA collected from the two leukemia cell lines are very similar and different with respect to the Raman spectrum of the DNA collected from normal cells. The differences are most visible in the 1000–1700 cm^−1^ spectral region. All the vibrational bands of leukemia DNA are more intense compared with normal DNA. For certain peaks, a maximum spectral shift of 20 cm^−1^ was measured.

**Figure 3:**
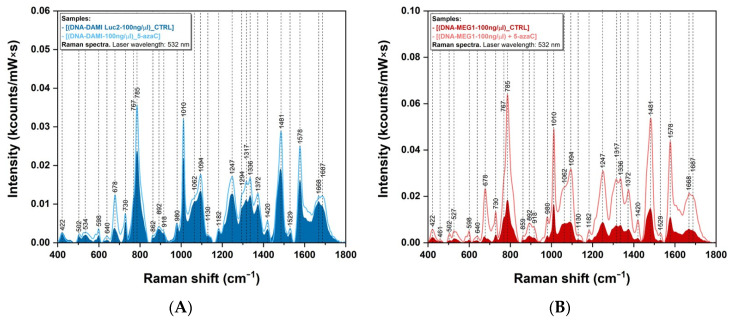
Raman spectra of DNA samples obtained from DAMI Luc2 (**A**) and MEG-1 (**B**) cell lines before (filled area) and after treatment with 5-azaC. The spectra were collected using a 532 nm laser.

The Raman spectra of DNA samples collected from leukemia cancer cells before and after treatment with 5-azaC are presented in Figure 3. After treatment, a significant increase in intensity for the vast majority of the peaks (678 cm^−1^, 785 cm^−1^, 1010 cm^−1^, 1094 cm^−1^, 1247 cm^−1^, 1317 cm^−1^, 1336 cm^−1^, 1481 cm^−1^, and 1578 cm^−1^) can be observed for both samples. The increase is more pronounced in the case of MEG-1 cells (Figure 3B) compared with DAMI Luc2 ones (Figure 3A). The position of the peaks is identical for both cell lines and no spectral shifts were observed after the treatment.

### 3.3. SERS Measurements

**Figure 4:**
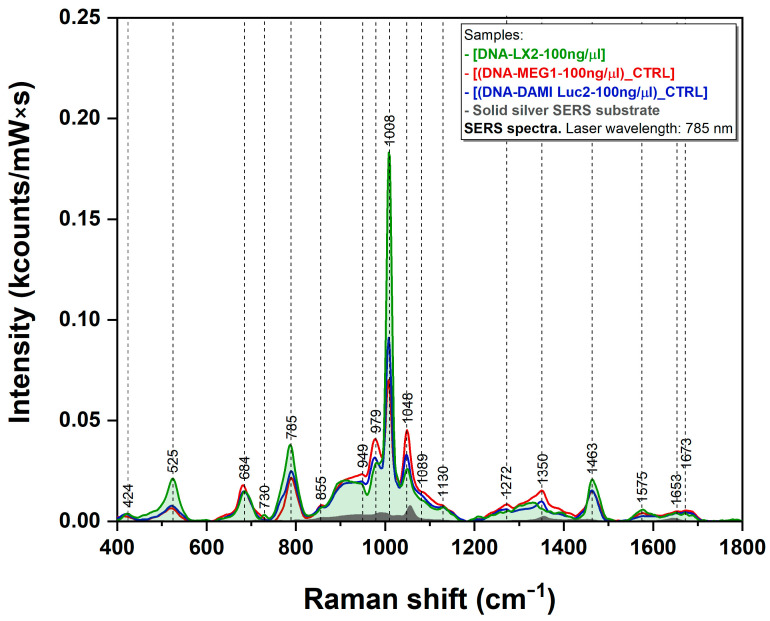
SERS spectra of DNA samples extracted from LX2 normal cell line (green spectrum) and DAMI Luc2 (blue spectrum) and MEG-1 (red spectrum) leukemia cell lines; recorded using a 785 nm laser. The grey spectrum represents the colloidal silver nanoparticles used as the substrate.

The DNA samples were mixed with concentrated and filtered silver colloidal nanoparticles. The solution was poured onto CaF_2_ glass and dried at 94 °C. The solid mixture, containing self-assembled plasmonic nanoparticles covered with ssDNA, was then analyzed by means of SERS. It turned out that this preparation method was able to generate very reproducible spectra, thus overcoming one of the major drawbacks encountered in SERS analysis of biological samples. The SERS spectra of DNA samples extracted from LX2 normal cells (green spectrum) and leukemia cells (DAMI Luc2—blue spectrum and MEG-1—red spectrum) are presented in Figure 4 together with the plasmonic substrate spectrum (in grey). Several vibrational bands can be clearly detected but one has to notice that all three spectra are dominated by the 1008 cm^−1^ band. Some of them are more intense in the case of normal DNA (525 cm^−1^, 785 cm^−1^, and 1008 cm^−1^), whereas others (979 cm^−1^, 1049 cm^−1^, and 1035 cm^−1^) are more amplified in the case of cancer cells. By far, the biggest difference can be noticed in the case of the 1008 cm^−1^ peak.

**Figure 5:**
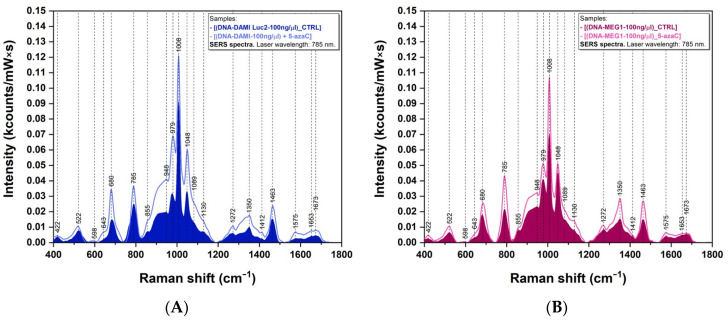
SERS spectra of DNA samples collected from DAMI Luc2 (**A**) and MEG-1 (**B**) cells before (filled area) and after treatment with 5-azaC; recorded using a 785 nm laser.

The treatment with 5-azaC led to an increase in intensity for most vibrational bands present in the SERS spectra of the two cancerous cell lines, as can be seen in Figure 5. These results are consistent with those obtained from the Raman analysis (Figure 3) presented above, proving the strong interaction of cancer cells with 5-azaC molecules. In the Supplementary Materials we included the SERS spectra of the four DNA bases (adenine—A, cytosine—C, thymine—T, and guanine—G) (Figure S1) and of 5mC (Figure S2) recorded using the same experimental conditions. We also included the SERS spectra of the colloidal silver nanoparticles used as a solid plasmonic substrate in Figure S3.

## 4. Discussion

The aim of our study was to investigate at the nanoscale the DNA samples collected from normal and leukemia cells by means of Raman/SERS in order to experimentally prove the differences existing in their methylation landscape. We also wanted to highlight the DNA demethylation induced by 5-azaC that was previously proposed in the literature [20,22,23]. In this regard, we introduced a facile and robust preparation method for the DNA samples as well as for the plasmonic substrates, allowing a reproducible Raman/SERS analysis with improved signal-to-noise ratios. 

The dsDNA melting process, performed for all DNA samples included in this study, leads to the formation of ssDNA through the breaking of hydrophobic stacking attractions between the bases without altering their composition. The heating treatment, performed at a temperature of 94 °C in a very similar manner to the one included in the RT-PCR analysis, allows a better investigation of the structural modifications occurring at the nucleobase level. Moreover, the interaction of ssDNA with purified anionic silver nanoparticles will occur mainly through nucleobases since the negatively charged deoxyribose moieties of ssDNA cannot interact with anionic silver nanoparticles as a direct consequence of their mutual electrostatic repulsion. 

In a very recent paper, Sina et al. showed that the different methylation landscapes of normal and cancerous epigenomes strongly impact their physicochemical and self-assembly properties in aqueous solutions and as they interact with solid plasmonic surfaces. Moreover, they have shown that normal genomic DNA has the tendency to create larger aggregates compared with those observed in the case of cancer DNA [39].

### 4.1. Raman Analysis

The Raman spectra presented in Figure 2 represent a strong experimental confirmation of this finding. As can be seen in the figure, the Raman spectra of cancer DNA are much richer in spectral features compared with normal DNA. The vibrational bands assigned to the four bases (A, C, T, and G) dominate the spectra. According to the literature [25,26,27,31,40], they are located at 728/1302 cm^−1^ (A), 783/1531 cm^−1^ (C), 749/1652 cm^−1^ (T), and 681/1713 cm^−1^ (G). These bands are all present in the case of cancer DNA (red and blue spectra). For normal DNA (green spectrum), the vibrational signatures of A are absent. Moreover, the wavenumbers of these bands are slightly shifted (less than 6 cm^−1^)compared with those reported in the literature, with the differences being higher in the case of normal DNA. These two observations can be explained only if one considers the much higher tendency of normal DNA to form aggregates, favoring a stronger interaction between the nucleobases.

On the other hand, the DNA sequences of leukemia cells are rich in purine bases and this fact is supported by the presence of several specific vibrational bands (675 cm^−1^ and 730 cm^−1^), assigned to G and A, respectively. In the Raman spectra (Figure 2), these peaks are much more intense in the case of cancer DNA with respect to normal DNA. It is well known that cancer cells are more prone to synthesize de novo nucleotides due to their intense metabolism [41,42] and this could be the explanation for the enhancement of these vibrational bands. Moreover, the adenine band, located at 730 cm^−1^ and clearly seen in our spectra only in the case of cancer DNA, has also been reported in other studies as being a possible marker of cancer [43,44].

Another very interesting spectral feature, supporting the different aggregation tendency of normal/cancer DNA induced by the methylation landscape, can be seen at 1094 cm^−1^ (red and blue curves in Figure 2). This band is assigned to localized stretching vibrations of the phosphate ($PO_{2}^{-}$) group belonging to the deoxyribose backbone. The intensity of this band is very high in the case of cancer DNA (no aggregation) but it is absent for normal DNA samples (strong aggregation). Deng et al. showed that the intensity of this band is almost the same in the case of different DNA samples [45], irrespective of base composition or sequence, so the only explanation that can be considered for its disappearance in the case of normal DNA is sample aggregation. A tentative assignment of the major vibrational bands presented in this study is summarized in Table S1 based on the scientific literature [31]. 

DNA methylation is a complex process with major influences on cell development. In certain conditions, it can lead to carcinogenesis [3,4,6]. DNA demethylation may be acquired using various inhibitor agents (azacytidine, decitabine, zebularine, and others) that can affect DNMTs enzymes’ group action. Some of these agents are currently approved for clinical practice [10]. One of the widely used demethylation agents is 5-azaC, which is an analog of cytosine. 5-azaC inhibits DNA methylation in leukemia cells by its incorporation in the DNA sequences during replication [6,12,24]. 

The effect of 5-azaC treatment on DAMI Luc2 and MEG-1 leukemia cell lines is presented in Figure 3. The addition of a 5-azaC demethylating agent on leukemia cells leads to an increase in the vibrational bands’ intensities for both cancer lines, in Raman (Figure 3) as well as in SERS (Figure 5). We suppose that this is a direct consequence of the fact that 5-azaC may be in competition with de novo synthesized cytosine pyrimidine bases for DNA incorporation [46]. By comparing the intensity of the vibrational bands, it turns out that the effect of 5-azaC treatment is more enhanced in the case of MEG1 cells (Figure 3B) compared with DAMI Luc2 (Figure 3A). No additional peaks were detected after the treatment with 5-azaC, neither in Raman nor SERS. These results can be explained if one considers that only 10–20% of 5-azaC deoxyribose is incorporated into DNA while 80–90% remains in its triphosphate configuration incorporated into RNA [20].

### 4.2. SERS Analysis

The ability of SERS to quantitatively analyze the evolution of small biomolecules present in biofluids as a result of their interaction with an external factor has been reported in the literature. For instance, Colceriu-Șimon et al. quantified, by means of SERS, the effect of low-dose irradiation on humans, proving its ability to detect an immediate increase in salivary thiocyanate and suggesting that this small molecule could be used as a valuable biomarker for the detection and identification of low-dose ionizing radiation effects [28].

Epigenetic reprogramming in cancer genomes creates a distinct methylation landscape encompassing clustered methylation at regulatory regions separated by large intergenic tracks of hypomethylated regions. Recently, it has been shown that the presence of a peak at 1008 cm^−1^ in the SERS spectra of cancer DNA, assigned to a rocking vibration of the methyl group of 5mC, can be considered a DNA methylation signature [40,44]. 

In this study, we performed SERS experiments on the DNA samples collected from normal and cancer cells. The SERS spectra of the three DNA samples included in this study are presented in Figure 4. The first striking observation that can be made is that all three spectra are dominated by the 1008 cm^−1^ vibrational band. To the best of our knowledge, this is the first paper that reports this behavior. Moreover, the intensity of this band is almost double in the case of normal DNA compared with cancer DNA. On the other hand, this difference is slightly visible in our Raman analysis as it can be seen in Figure 2.

Li et al. reported the occurrence of two additional Raman peaks in the 1200–1700 cm^−1^ region as a direct consequence of DNA methylation. These peaks, located at 1239 cm^−1^ and 1639 cm^−1^, are assigned to C-N stretching mode and C=O stretching mode of the aromatic ring, respectively. By carefully analyzing the Raman spectra presented in Figure 2, one can observe that these peaks are very intense in the case of cancer DNA compared with normal DNA. However, in the case of SERS, the intensities of these two peaks are identical for normal and cancer DNAs. The only explanation for this observation must be related to the geometry of interaction between 5mC and C and the plasmonic substrate, having in mind that the geometry of interaction has a major influence on the SERS spectra of the adsorbed molecules in accordance with the surface selection rules developed by Moskovits [47]. In a previous paper, we showed that in the case of SERS experiments performed on silver colloids, the molecules have the tendency to adsorb on the surface of silver nanoparticles through the lone electron pairs of N and O atoms [48]. Sancho-Cortez et al. performed a complete Raman/SERS analysis of C and 5mC and concluded that the adsorption of C and 5mC on silver nanoparticles occurs through the carbonyl group [49]. If one considers that the methyl group, lying in very close vicinity of the plasmonic surface, does not form any coordination bond with metals, the intensity of the 1008 cm^−1^ vibrational band, assigned to a rocking vibration of methyl group, can be explained. As such, one could consider that under proper experimental conditions, the 1008 cm^−1^ peak can be regarded as a valuable DNA methylation signature. 

The here-proposed method for SERS analysis favors the interaction of the bases present in ssDNA with the plasmonic nanoparticles acting as SERS substrates. The disappearance of the 1094 cm^−1^ band, assigned to a localized stretching vibration of the phosphate ($PO_{2}^{-}$) group belonging to the deoxyribose backbone, highly visible in the Raman spectra, is a further proof of this geometry of interaction. If one considers that SERS has the capacity to strongly amplify the vibrational bands of the molecules present in the nanoscale vicinity of the plasmonic substrates, the very high intensity of the 1008 cm^−1^ peak represents a strong experimental proof of a uniform distribution of 5mC in the normal DNA samples. This is not the case for cancer samples, where hypermethylated regions form, especially at the CpG sites. This variable distribution of 5mC leads to a strong decrease in the intensity of the 1008 cm^−1^ band, as can be seen in Figure 4 (red and blue curves).

## 5. Conclusions

We investigated, by means of Raman/SERS, DNA samples collected from normal (LX2) and leukemia cancer cells (DAMI Luc2 and MEG-1). Raman experiments were performed on ssDNA samples generated using a protocol similar with the one used in RT-PCR analysis. The SERS experiments were performed by incubating the DNA sample with in-house-synthesized silver nanoparticles, concentrated, and purified by means of the tangential flow filtration (TFF) method, followed by a heat treatment at 94 °C in order to facilitate the interaction of DNA bases with the plasmonic nanoparticles. The effect of treatment with a demethylating agent (5-azaC) on DAMI Luc2 and MEG-1 cell lines was also investigated by Raman/SERS analysis. Our results showed that Raman analysis allows the discrimination between cancer and normal ssDNA. This spectral discrimination is based on a much stronger tendency of normal genomic DNA to create larger aggregates compared with those observed in the case of cancer DNA, as a direct consequence of a different methylation landscape. The SERS analysis performed on the same samples identified a highly intense vibrational peak (1008 cm^−1^) that, under proper experimental conditions, can be regarded as a valuable DNA methylation signature. In the case of DAMI Luc2 and MEG-1 leukemia cell lines treated with 5-azaC, an increase in the vibrational bands’ intensities for both cancer lines was detected. No additional bands were observed in our Raman and SERS spectra. These results can be explained if one considers that only a small fraction of 5-azaC deoxyribose is incorporated into DNA while the rest remains in its triphosphate configuration incorporated into RNA. As a future perspective, the combined Raman/SERS analysis presented in our work might be applied to other DNA samples in order to evaluate the distinct methylation landscape induced by epigenetic reprogramming in different cancer genomes.

## Data Availability

Not applicable.

## Supplementary Materials

The following supporting information can be downloaded at: https://www.mdpi.com/article/10.3390/s23010346/s1, Figure S1: SERS spectra of Adenine (a), Cytosine (b), Thymine (c), and Guanine (d) recorded using a 785 nm excitation laser; Figure S2: SERS spectra of 5-methylCytosine recorded using a 785 nm excitation laser; Table S1: DNA Raman and SERS peaks assignments. References [50,51] are cited in the supplementary materials.
