# Supplementary material for: Nanoscale Investigation of DNA Demethylation in Leukemia Cells by Means of Ultrasensitive Vibrational Spectroscopy
Source: Sensors (Basel). 2022 Dec 29;23(1):346. doi: 10.3390/s23010346 (PMC9823440; doi:10.3390/s23010346)
Supplement: Supplementary file 1 [file sensors-23-00346-s001.zip › sensors-2094158-supplementary.pdf]

Supplementary Materials

Nanoscale Investigation of DNA Demethylation in Leukemia Cells by Means of Ultrasensitive Vibrational Spectroscopy

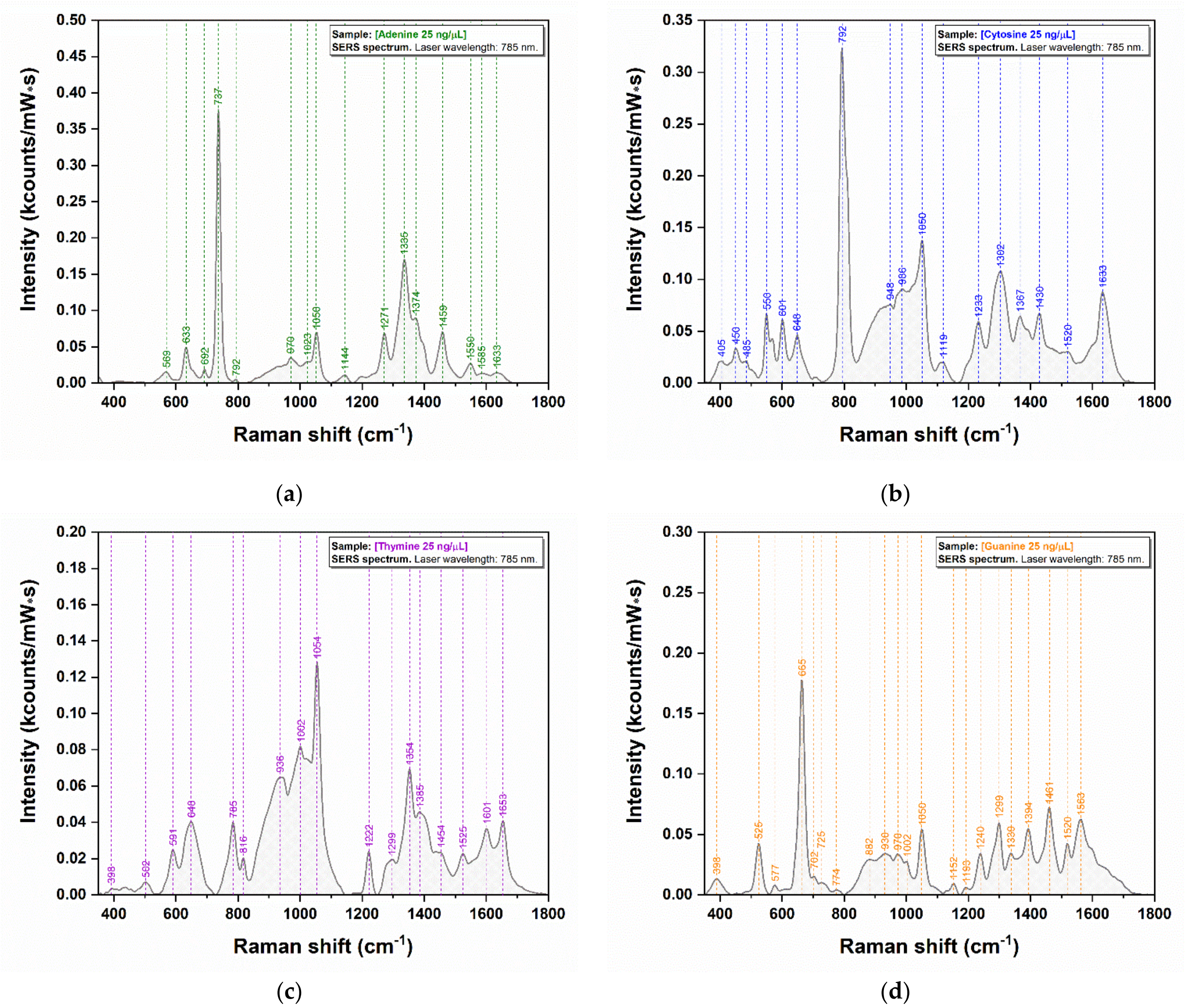

Figure S1. SERS spectra of Adenine (**a**), Cytosine (**b**), Thymine (**c**) and Guanine (**d**) recorded using a 785 nm excitation laser.

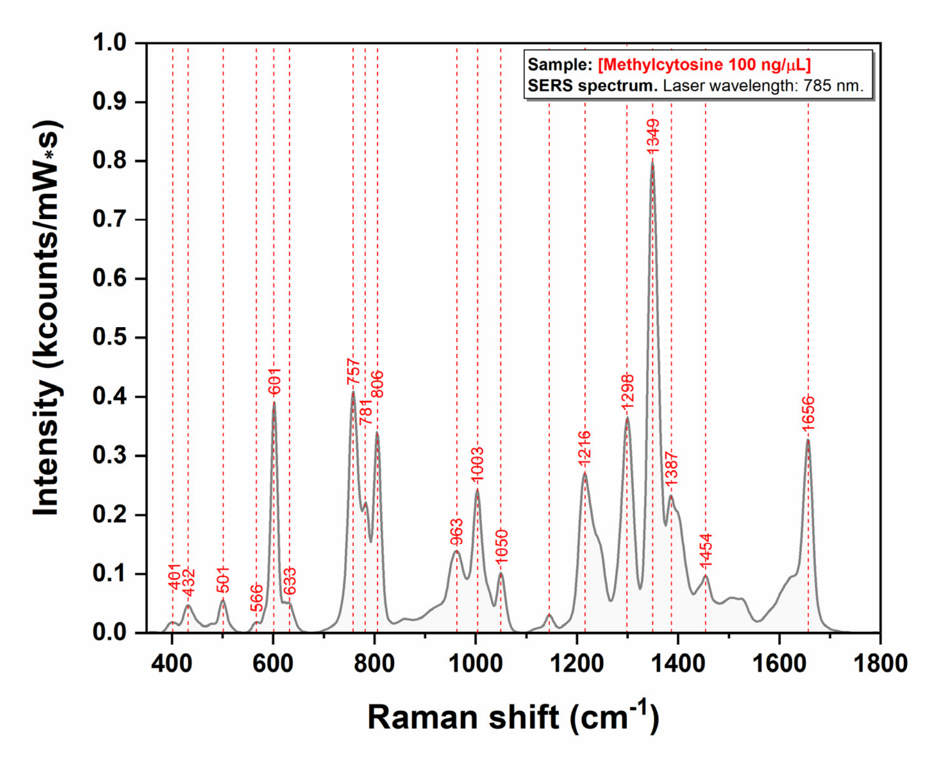

Figure S2. SERS spectra of 5-methylCytosine recorded using a 785 nm excitation laser.

Table S1. DNA Raman and SERS peaks assignments.

RAMAN Wavenumber (cm⁻¹)	SERS Wavenumber (cm⁻¹)	ASSIGNAMENTS		References
422	424	Thymine	Ring out-of-plane	[1–3]
461		Deoxyribose phosphate backbone		[4]
502		Thymine, Guanine	G: ($C_2N_1C_6^b$+N_9R^b − $C_5C_4N^b$)	[2,4]
527		N-Acetyl-D-asmine, Citric acid		[3]
598		Cytosine		[2–4]
638		Adenine, Cytosine		[4]
675	684	Guanine, Cytosine, Glutathione	In-phase ring stretching of the six-membered ring except C_4C_5	[2,4–7]
730	730	Adenine	Ring stretching	[2–7]
767		Thymine, Fumarate, L-Tryptophan		[3]
785	785	Thymine, Cytosine, Citric acid, Phosphoenolpyruvate	Ring breathing	[1–4,6]
859	855	Thymine, Guanine	T: C=O bending<br>G: -$N_7C_5^s$-$N_1C_2N_3^b$	[1–3]
890		Deoxyribose phosphate backbone		[4]
945	949	Adenine	NH_2^r+$N_1C_6^s$	[2,3]
980	979	Thymine		

995		Cytosine, Deoxyribose phosphate backbone		[3,4]
1008	1008	Thymine, Deoxyribose phosphate backbone, 5-Methylcytosine	Ring stretch	[1,4,7]
1049	1048	Thymine, Guanine	Methyl twist	[1,3]
1062		D-(C)-Galactosamine		[3]
1094	1089	Deoxyribose phosphate backbone		[4,6]
1130		Ascorbic acid		[3]
1182		Thymine, Cytosine	T: $-C_6^bH+C_2N_3^s$	[2,4]
1247		Adenine, Thymine		[3,4]
	1272	Thymine, Guanine	T: $Ring^s+CH^b$<br>G: $-C_8N_7^s-N_1C_6^s+N_7C_5^s$	[1,2]
1294		Cytosine	$N_1C_6^s+C_5C_6^s$	[2,4]
1317		Guanine		[4]
1334		Adenine	$-N_7C_5^s+C_8N_7^s$	[2–4,6]
	1350	Adenine		[4]
1372		Adenine, Thymine, Guanine	T: C–H + Methyl bend	[1,3,4]
1402		Adenine	$-N_1C_6^s+C_6N_{12}^s$	[2]
1420		Thymine, Cytosine, Guanine	N–H bend	[1–4]
1465	1463	Adenine, Thymine, Cytosine, Guanine, Deoxyribose phosphate backbone, Palmitic acid, 14-Methylpentadecanoic		[3,4]
1481		Adenine, Thymine, Cytosine, Guanine	A: $C_2H^b-N_1C_2^s+N_3C_2^s$<br>C: $N_1C_6^s+N_3C_4^s$<br>G: $N_1C_2^s-C_4N_9^s$	[2–4,6]
1529		Cytosine	C: $-N_3C_4^s-N_1C_2^s$	[2–4]
1578	1575	Adenine, Guanine	Ring stretch<br>G: $N_3C_4^s-C_4C_5^s$	[2,4–6]
1603		Cytosine, Guanine	G: $N_3C_4^s-C_4C_5^s$	[2–4]
	1653	Thymine, Cytosine, H_2O		[3–5]
1668	1673	Thymine, Guanine	G: $C_6=O^s-C_5C_6^s$	[1–4]
1687		Cytosine		[3]

r = rocking; s = stretching; b = bending

References

1. Aroca, R.; Bujalski, R. Surface enhanced vibrational spectra of thymine. *Vib. Spectrosc.* **1999**, *19*, 11–21, doi:10.1016/S0924-2031(99)00003-X.

2. Otto, C.; van den Tweel, T.J.J.; de Mul, F.F.M.; Greve, J. Surface-enhanced Raman spectroscopy of DNA bases. *J. Raman Spectrosc.* **1986**, *17*, 289–298, doi:10.1002/jrs.1250170311.

3. De Gelder, J.; De Gussem, K.; Vandenabeele, P.; Moens, L. Reference database of Raman spectra of biological molecules. *J. Raman Spectrosc.* **2007**, *38*, 1133–1147, doi:10.1002/jrs.1734.

4. Prescott, B.; Steinmetz, W.; Thomas, G.J. Characterization of DNA structures by laser Raman spectroscopy. *Biopolymers* **1984**, *23*, 235–256, doi:10.1002/bip.360230206.

5. Garcia-Rico, E.; Alvarez-Puebla, R.A.; Guerrini, L. Direct surface-enhanced Raman scattering (SERS) spectroscopy of nucleic acids: from fundamental studies to real-life applications. *Chem. Soc. Rev.* **2018**, *47*, 4909–4923, doi:10.1039/C7CS00809K.

6. Barhoumi, A.; Zhang, D.; Tam, F.; Halas, N.J. Surface-Enhanced Raman Spectroscopy of DNA. *J. Am. Chem. Soc.* **2008**, *130*, 5523–5529, doi:10.1021/ja800023j.

7. Moisoiu, V.; Stefancu, A.; Iancu, S.D.; Moisoiu, T.; Loga, L.; Dican, L.; Alecsa, C.D.; Boros, I.; Jurj, A.; Dima, D.; et al. SERS assessment of the cancer-specific methylation pattern of genomic DNA: towards the detection of acute myeloid leukemia in patients undergoing hematopoietic stem cell transplantation. *Anal. Bioanal. Chem.* **2019**, *411*, 7907–7913, doi:10.1007/s00216-019-02213-2.
